# Letter from J. D. Hunter, M. D., New Orleans, La.

**Published:** 1890-06

**Authors:** J. D. Hunter

**Affiliations:** 352 Tulane Avenue; New Orleans


NEW ORLEANS, May 12, 1890.

*Editors Atlanta Medical and Surgical Journal:*

DEAR SIR—To answer fully and specifically the great number of communications received from physicians throughout the country, asking additional information in relation to the use of *Potassii Nitras* in malarial affections, would necessitate the occupation of the greater part of my time. I will say, in brief, that I have treated more than two hundred cases of chronic chills of malarial origin, from a few months to years' standing; many complicated with enlargement of the liver and spleen, dropsy, jaundice, etc., all more or less emaciated and anemic. Nearly every case was cured with a single dose of *Potassii Nitras.* From two to fifteen grains of the salt, according to age, dissolved in a half ounce of water, administered just prior to a chill or during its continuance, did not only abort or arrest the chill, but effectually prevented its recurrence.

In order to test the value of the remedy, I employed no subsequent treatment, but left the restoration to health (which was in nearly every instance rapid and satisfactory) to the *vis medicatrix naturæ.*

I am fully assured that from two to fifteen grains of *Potassii Nitras* will usually abort or arrest a chill arising from any cause. A large dose is not well borne by the stomach, and frequently, in my hands, caused most alarming and distressful symptoms, by producing a prolonged depression of the heart's action. I am

Most respectfully,

352 *Tulane Avenue.* J. D. HUNTER, M. D.

